# Comparison of Anterior Capsule Polishing on the Rate of Neodymium: YAG Laser Capsulotomy After Two Multifocal Intraocular Lens Implantation

**DOI:** 10.3389/fmed.2022.815966

**Published:** 2022-03-10

**Authors:** Lin Leng, Huiran Bai, Honglei Li, Dongle Liu, Yanfeng Han, Xiaoming Wu

**Affiliations:** ^1^Qingdao Eye Hospital of Shandong First Medical University, Qingdao, China; ^2^State Key Laboratory Cultivation Base, Shandong Provincial Key Laboratory of Ophthalmology, Eye Institute of Shandong First Medical University, Qingdao, China

**Keywords:** anterior capsule polishing, Nd: YAG laser capsulotomy, multifocal intraocular lens, cataract, posterior capsule opacification

## Abstract

**Purpose:**

To compare the impact of anterior capsule polishing (ACP) during cataract surgery on the rate of neodymium: YAG (Nd: YAG) laser capsulotomy in pseudophakic eyes with two multifocal intraocular lenses (MIOLs).

**Methods:**

Data were collected on patients who underwent cataract surgery and implanted segmental refractive MIOLs (SBL-3, Lenstec) or diffracted MIOLs (AT LISA tri 839MP, Carl Zeiss Meditec). The participants were divided into ACP and non-ACP groups based on whether the anterior capsule was polished. The primary outcome measure was whether Nd: YAG capsulotomy was performed during the 3 years follow-up. We used Kaplan–Meier survival curves to determine the time from IOL implantation to Nd: YAG laser capsulotomy.

**Results:**

ACP and non-ACP groups comprised 70 and 60 eyes, respectively. One year postoperatively, 7.14% of ACP group eyes and 8.33% of non-ACP group required Nd: YAG laser capsulotomy (*P* > 0.99). After 2 years, it was 24.29 and 18.33%, respectively (*P* = 0.52), while after 3 years, it reached 30.0 and 28.33% (*P* = 0.85). No distinct difference existed in the probability of using Nd: YAG laser in both groups evaluated using Kaplan-Meier survival curves (*P* = 0.81). Patients with diffractive MIOLs (AT LISA tri 839MP) implantation were more likely to require Nd: YAG laser capsulotomy (*P* < 0.01).

**Conclusion:**

Polishing the anterior capsule had no remarkable effect on reducing the rate of Nd: YAG laser capsulotomy following phacoemulsification in MIOLs. Patients with diffractive MIOLs implantation had a high probability of requiring Nd: YAG laser capsulotomy.

## Introduction

Posterior capsule opacification (PCO) is the most frequent long-term adverse event following cataract surgery, resulting in visual impairment and requiring additional surgery (1). While PCO can be treated by cutting a hole in the dorsal capsule using Nd: YAG laser, this process might cause other unintended consequences, such as raised intraocular pressure (IOP), corneal haze, uveitis, intraocular lens (IOL) pits, cystoid macular edema, and retinal detachment (2).

Numerous studies have assessed the pathogenicity of PCO to establish potential prevention methods (3–5). Capsular opacification is caused by the proliferation, migration, and transdifferentiation of lens epithelial cells (LECs) that are usually located on the interior of the frontal capsule and remain there following cataract operation (3, 4). These LECs attempt to differentiate or undergo epithelial-mesenchymal transformation, creating different kinds of cell groups in the posterior capsule and resulting in lens capsule contraction and fibrosis (4, 5). Therefore, removing residual LECs could reduce the likelihood of PCO development.

The IOL evolved and developed from a traditional monofocal IOL to a multifocal intraocular lens (MIOLs) for improved near vision. Patients receiving MIOLs have high expectations for their postoperative vision and may require IOL exchange due to various causes (6). Patients with MIOLs appear to be more susceptible to PCO than those with monofocal IOLs (7). No conclusive evidence previously existed regarding the influence of anterior capsule polishing (ACP) on the PCO of monofocal IOLs, and studies on the impact of MIOLs are scarce. We aimed to explore the impact of ACPon MIOLs and whether this impact differed for different effects for different MIOL design types. In this study, we retrospectively evaluated ACP’s impact on PCO and the requisites for Nd: YAG laser capsulotomy after the implantation of two different designs of MIOLs.

## Patients and Methods

This 3-year retrospective consecutive case-series study was conducted at Shandong Eye Institute, Qingdao Eye Hospital. We mined and collected medical data from the hospital’s patient files. A clinical research assistant supervised the anonymous completion of a form. The study’s protocol and measurements were in line with the tenets of the Declaration of Helsinki. The study followed appropriate guidelines and was approved by the Qingdao Eye Hospital committee (ChiCTR1800015251). The inclusion criteria for patients were as follows: being of an age greater than 18 years; having pre-operative and post-operative corneal astigmatism within?1.00 D and axial length between 22.0 and 24.5 mm; and giving written informed consent for participation in this study. Exclusion criteria were as follows: having intra- and postoperative complications; having previous ocular trauma; and having ocular pathologies such as glaucoma, diabetes mellitus, complicated cataracts, progressive retinopathy, uveitis, and previous ocular surgery.

Using electronic medical records, a list of patients who underwent cataract surgery and the implantation of segmental refractive MIOL (SBL-3, Lenstec, Inc., Christ Church, Barbados) or diffracted MIOL (AT LISA tri 839MP, Carl Zeiss Meditec, Jena, Germany) from May 2016 to April 2017 was collected. Table 1 summarizes the specific features of the two MIOLs designs. We reviewed all corresponding medical records and included patients who met the criteria. All relevant pre- and post-data before and after surgery were checked and extracted by the end of April 2020.

**TABLE 1:** Characteristics of the two multifocal intraocular lens.

Parameters	AT LISA tri 839MP	SBL-3
Optical diameter, mm	6.00	5.75
Length, mm	11.0	11.0
Diopter range, D	0 to +32.0	+10.0 to +36.0
Add power, D	3.33	3.00
Material	Hydrophilic acrylic with hydrophobic surface (25%)	Hydrophilic acrylic
Construcion	1 piece	1 piece
Haptic style	Plate-haptic	Closed-loop
Optic type	A central trifocal zone over a diameter of 4.34 mm, and a peripheral bifocal zone from 4.34 to 6 mm	Bi-aspheric, neutral aberration
A-constant	118.6	118.4

Patients were categorized into two groups, ACP and non-ACP, based on their operation mode. The decision to perform ACP was based on the amount of visible residual LECs. The surgeon was unaware that relevant data could be used later in this comparative study. An experienced ophthalmologist (WXM) conducted all operations under topical anesthesia. The ophthalmologist made a precise 2.2 mm corneal incision, followed by a 5–5.5 mm continuous circular capsulorhexis (CCC), phacoemulsification, and irrigation and aspiration of the cortex. A segmental refractive MIOL or a diffractive MIOL was then put into a capsular bag. In the ACP group, undersurface polishing of the anterior capsule was done in all quadrants and at all clock hours within the visible range using a polisher (MR-I117-2, Suzhou Mingren Medical Apparatus and Instruments Co., Ltd., Suzhou, China).

All patients received topical prednisolone (1.0% eye drops, four times daily, in a tapering dose for 4 weeks), topical levofloxacin (0.5% eye drops four times daily for 2 weeks), and sodium bromfenate (0.1% eye drops, two times daily for 4 weeks). Both postoperative uncorrected distance visual acuity (UDVA) and best-corrected visual acuity (BCVA) were recorded in logMAR units during the follow-up period. Our primary endpoint was the incidence of Nd: YAG laser capsulotomy. The secondary endpoint was the time at which Nd: YAG laser capsulotomy was performed after cataract surgery. We defined PCO as any central opacity or wrinkling of the posterior capsule on slit lamp examination. Indications for Nd: YAG laser capsulotomy were similar for the two groups, based on the following visual acuity and clinical signs: proximal or distal visual acuity decreased by two lines and confirmed by PCO during a clinical examination.

The data were statistically analyzed using IBM SPSS Statistics version 22.0 (IBM Corp., New York, NY, United States). Group comparisons at the time of intervention were assessed using the chi-square or Fisher exact test for qualitative data and an independent *t*-test for quantitative data. We utilized Kaplan–Meier probability curves to determine the risk of Nd: YAG laser capsulotomy in a certain period following surgery. A logarithmic rank test was employed to compare two groups of probability curves. Differences with a *P*-value of 0.05 or less were considered statistically significant.

## Results

This study enrolled 121 patients (130 eyes). Among them, 66 were male, and 55 were female. The ACP and non-ACP groups comprised 70 and 60 eyes, respectively. Table 2 summarizes the characteristics of the patients and their corresponding ocular parameters. There were no remarkable differences in preoperative patient characteristics between the ACP and non-ACP groups, such as age, grade of lens nuclearity, lens thickness, anterior chamber depth, and axial length.

**TABLE 2:** Preoperative patient characteristics.

Parameter	Group		*P*-value
	ACP	Non-ACP	
Patients/Eyes (n)	65/70	56/60	–
Male/Female (n)	34/31	32/24	0.71^+^
Age (years)	50.20 ± 11.03	53.13 ± 13.14	0.17*
Eye laterality (right/left)	41/29	32/28	–
Grade of lens nuclear	3.01 ± 0.66	3.24 ± 0.97	0.77*
Lens thickness (mm)	2.93 ± 0.18	2.89 ± 0.25	0.91*
Corneal power (D)	43.11 ± 1.76	42.48 ± 1.91	0.87*
ACD (mm)	3.12 ± 0.26	3.14 ± 0.19	0.92*
AL (mm)	23.49 ± 0.90	23.52 ± 0.91	0.85*

ACP, anterior capsule polishing; ACD, anterior chamber depth; AL, axial length; ^+^chi-square test; * independent t-test.

Refraction, UDVA, and BCVA measurements were assessed postoperatively at 1, 2, and 3 years after surgery (Table 3). If patients had undergone the Nd: YAG capsulotomy at the time of the follow-up examination, they were not included in the analysis. Table 4 displays the characteristics of patients who underwent Nd: YAG capsulotomy during the follow-up period. The ACP and non-ACP groups consisted of 21 and 17 eyes operated on using the Nd: YAG capsulotomy approach. Before Nd: YAG capsulotomy, no significant difference was found in the UDVA (0.42 ± 0.22 log MAR vs. 0.49 ± 0.42 log MAR, *P* > 0.05) and BCVA (0.31 ± 0.38 log MAR vs. 0.35 ± 0.40 log MAR, *P* > 0.05) between the ACP group and non-ACP group (Table 4). Both UDVA and BCVA significantly improved after Nd: YAG capsulotomy, however, there was still no statistical between-group difference in UDVA (0.13 ± 0.41 log MAR vs. 0.15 ± 0.20 log MAR, *P* > 0.05) and BCVA (0.07 ± 0.19 log MAR vs. 0.08 ± 0.27 log MAR, *P* > 0.05). One year postoperatively, 7.14% of eyes in the ACP group and 8.33% in the non-ACP group required Nd: YAG laser capsulotomy (*P* > 0.99). After 2 years, these numbers were 24.29% and 18.33%, respectively (*P* = 0.52), and after 3 years, they were 30.0 and 28.33% (*P* = 0.85).

**TABLE 3:** Visual acuity and refractive outcomes of all patients.

		Postoperative		
Parameter	Preoperative	1 Year	2 Year	3 Year
SE (D)	−1.32 ± 1.64	−0.05 ± 0.83	−0.06 ± 0.95	0.06 ± 0.80
**Visual acuity (log MAR)**				
UDVA	0.59 ± 0.38	0.16 ± 0.10	0.14 ± 0.10	0.13 ± 0.14
BCVA	0.41 ± 0.27	0.09 ± 0.08	0.09 ± 0.11	0.10 ± 0.09

*SE, spherical equivalent; UDVA, uncorrected distance visual acuity; BCVA, best corrected visual acuity.*

**TABLE 4:** Characteristics of patients who underwent of Nd: YAG capsulotomy during the follow-up period.

Parameter	Group		*P*-value
	ACP	Non-ACP	
Eyes (n)	21	17	−
Mean time (months)*	18.05 ± 6.50	19.06 ± 11.45	0.74#
UDVA (log MAR)	0.42 ± 0.22	0.49 ± 0.42	0.50#
BCVA (log MAR)	0.31 ± 0.38	0.35 ± 0.40	0.81#
**Nd:YAG rate (%)**			
1 year	7.14	8.33	> 0.99^+^
2 years	24.29	18.33	0.52^+^
3 years	30.0	28.33	0.85^+^

ACP, anterior capsule polishing; UDVA, uncorrected distance visual acuity; BCVA, best-corrected visual acuity; # independent t-test; *days from surgery to Nd:YAG; + chi-square test.

Table 5 illustrates the distribution of patients implanted with segmental refractive and diffractive MIOLs who underwent Nd: YAG laser capsulotomy during the 3-year follow-up period. All segmental refractive MIOL implantation procedures that required Nd: YAG laser capsulotomy occurred within the first 2 years after cataract surgery.

**TABLE 5:** Neodymium: YAG laser capsulotomies performed in the 3-year follow-up examination.

IOL	Group	Years		
		1	2	3
Segmental refractive MIOL (*n* = 41)	ACP	1	4	0
Diffractive MIOL (*n* = 29)	ACP	4	8	4
Segmental refractive MIOL (*n* = 27)	Non-ACP	3	1	0
Diffractive MIOL (*n* = 33)	Non-ACP	2	5	6

*IOL, intraocular lens; MIOL, multifocal intraocular lens; ACP, anterior capsule polishing.*

**FIGURE 1:**
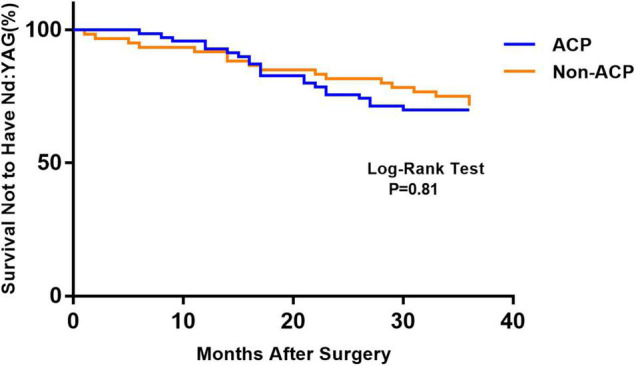
Kaplan–Meier survival plots for eyes by surgical treatment (Nd:YAG, neodymium:YAG laser capsulotomy; ACP, anterior capsule polishing).

**FIGURE 2:**
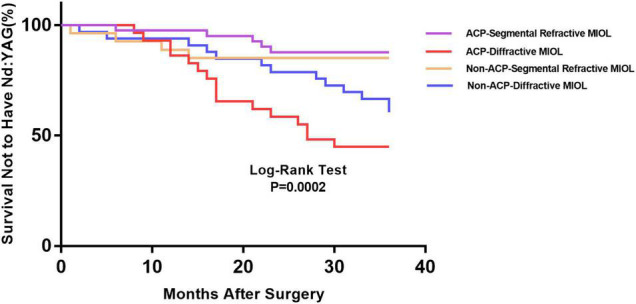
Kaplan–Meier survival plots for eyes by different IOL types in different surgical treatments (Nd:YAG, neodymium:YAG laser capsulotomy; ACP, anterior capsule polishing).

**FIGURE 3:**
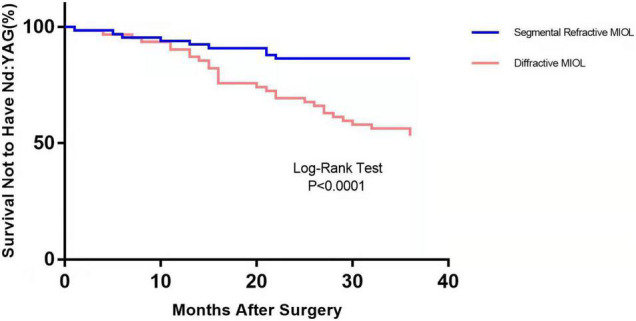
Kaplan–Meier survival plots for eyes by different IOL types (Nd:YAG, neodymium:YAG laser capsulotomy).

**FIGURE 4:**
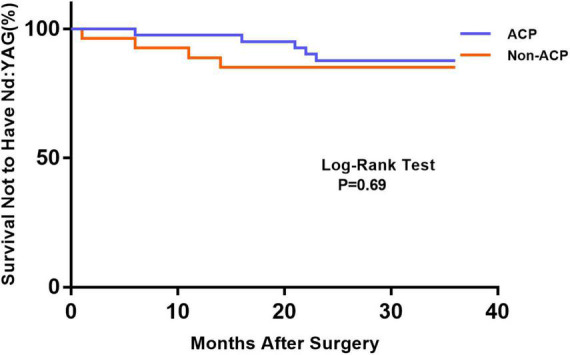
Kaplan–Meier survival plots for eyes by different surgical treatments about segmental refractive MIOL (SBL-3) (Nd:YAG, neodymium: YAG laser capsulotomy; ACP, anterior capsule polishing).

**FIGURE 5:**
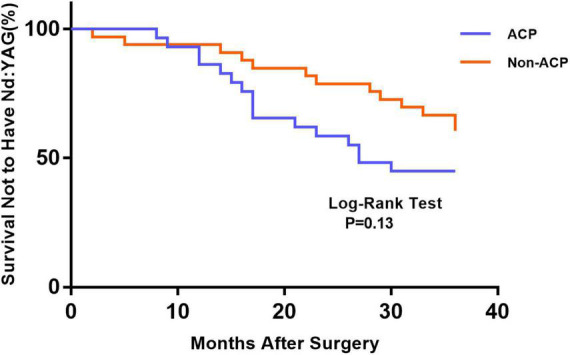
Kaplan–Meier survival plots for eyes by different surgical treatments about diffractive MIOL (AT LISA tri 839MP) (Nd:YAG, neodymium:YAG laser capsulotomy; ACP, anterior capsule polishing).

Figure 1 depicts the survival curves for the percentage of patients who did not require Nd: YAG laser therapy as a function of time. The survival curves demonstrated no remarkable difference in the probability of receiving Nd: YAG laser treatment between the ACP and non-ACP groups (*P* = 0.81). Based on the kind of IOL implanted, we conducted an analysis of the survival curves by mode of operation using Kaplan-Meier. Consequently, statistical differences were observed (*P* = 0.0002, Figure 2). Patients with implantation of diffractive MIOLs (AT LISA tri 839MP) were more likely to require Nd: YAG laser capsulotomy (*P* < 0.0001, Figure 3). There was no statistically significant difference between patients with segmental refractive MIOL implantation, with or without ACP (*P* = 0.69, Figure 4). Moreover, no marked difference was observed between patients with diffractive MIOL implantation, as shown in Figure 5 (*P* = 0.13).

## Discussion

In the past two decades, the use of ACP to prevent PCO after uneventful phacoemulsification has been a frequent topic of discussion and research among cataract surgeons worldwide (8–12). Nevertheless, no consensus has been reached on the efficacy of polishing. To our knowledge, this is the first study to investigate the effect of ACP on the rate of Nd: YAG laser capsulotomy for different designs of MIOL. According to this retrospective analysis, polishing the anterior capsule had no remarkable effect on reducing the need for Nd: YAG laser capsulotomy; however, patients with diffractive MIOL (AT LISA tri 839MP) implantation had a high probability of requiring Nd: YAG laser capsulotomy.

No significant difference in the rate of Nd: YAG capsulotomy was found between non-polishing and polishing groups 3 years postoperatively. The current research findings indicate that the effect of ACP on the rates of PCO and Nd: YAG capsulotomy remains inconclusive. A meta-analysis of studies revealed that the rate of PCO was reduced in the ACP group based on the summary odds ratio of the PCO rate (OR: 0.42; 95% CI: 0.24–0.73) and that ACP improved visual function (12). Sacu et al. (13) hypothesized that lower anterior capsule opacification (ACO) and fibrotic PCO with both round-edged silicone IOLs 2 years postoeratively in eyes with extensively polished anterior capsules. Other articles about ACP found that it did not affect PCO (14–18). A 3-year randomized trial revealed that ACP did not prevent PCO formation but enabled the formation of more regeneratory cataracts (14). Sachdev et al. (15) demonstrated that PCO incidence in a 360-degree polishing study group was lower but not markedly different at a 1-year follow-up. Consistent with this finding, a different study revealed no apparent advantages of scraping on ACO development in a cohort of 120 eyes at a 6 month follow-up (16). Liu et al. (18) explained why polishing the anterior capsule did not reduce PCO rates: surgical techniques, including ACP, had a crucial effect on residual cell growth. Notably, although the cells under the anterior capsule were almost entirely removed by polishing before culturing, ACP significantly promoted the growth of pouch cells cultured during phacoemulsification. Capsule polishing did not eliminate all LECs but stimulated the strong proliferation of remaining cells, whereas numerous living cells tended to die in unpolished eyes, leading to decreased proliferation. When Menapace et al. (14) prospectively analyzed YAG rates after 3 years, they discovered that 54% of eyes that received ACP required Nd: YAG capsulotomy compared to only 36% of eyes without ACP. The exact mechanism by which PCO is initiated is still unknown. Additional research is required to elucidate the physiology of PCO and the mechanism of barrier formation at the IOL optic barrier.

In the present study, we employed Kaplan–Meier survival curves primarily to analyze the rate of Nd: YAG capsulotomies between the two groups. We found statistical differences between the two groups based on the type of IOL implanted. In the ACP and non-ACP groups, we found that diffractive MIOLs (AT LISA tri 839MP) produced a higher incidence of PCO than segmental refractive MIOLs (SBL-3). It has previously been found that hydrophobic IOLs could reduce the incidence of PCO compared with hydrophilic IOLs (19). Both optics are hydrophilic materials, but the surface of AT LISA tri MIOL is coated with hydrophobic materials, which should theoretically result in better PCO inhibition performance. However, our study did not support this conclusion. Optic edge profile and haptic design could also affect PCO occurrence. Nováček et al. (20) compared two diffractive IOLs of the same material and discovered that the percentage of eyes undergoing Nd: YAG capsulotomy for PCO was significantly higher in the AT LISA tri group than in the Liberty (Medicontur Medical Engineering Ltd., Zsámbék, Hungary) group. They speculated that differences in haptic design might result in remarkable differences in PCO severity between groups (20). Sacu et al. (13) suggested that residual LECs left in the capsular bag may invade the retro-optical capsule through the optic-edge barrier. The oversized haptics stretch the capsular bag into an oval shape, inducing stress folds and fusion along the IOL axis, as well as incomplete capsule closure, resulting in barrier failure and invasion by LECs (21). Compared to solid haptic, closed-loop haptics are more flexible and thus easier to deform under the compressive force of the capsular bag, causing the bag to fit better. The difference in haptic design could account for the differences observed in Nd: YAG capsulotomy rates between the two types of MIOLs.

With the continuous improvement of IOL design technology, new MIOLs are designed to enable patients to achieve independence from glasses. Modern MIOLs use a non-physiological optic method of refracting light to create multiple focal points and facilitate a solution to presbyopia, which is more susceptible to PCO occurrence (22). Previous studies have focused more on ACP’s impact on the rate of PCO or Nd: YAG capsulotomy in patients with monofocal IOLs (13–18). In this study, we demonstrated that ACP had no impact on PCO formation in patients with MIOLs. A recent study recommended ACP for eyes only with a higher risk of anterior capsule contraction, such as those with myotonic dystrophy and high myopia, and those requiring a peripheral retinal examination following surgery (23). As a result, ophthalmologists may not routinely use intraoperative ACP to reduce PCO incidence. However, the role of ACP in maintaining the stability of IOLs cannot be ignored, particularly for MIOLs, as some studies have demonstrated that ACP reduces the occurrence of ACO and anterior capsule contraction (12, 24, 25).

Some limitations of our study should be considered. First, study was not randomized because implanted MIOLs were selected according to patients’ requirements for intermediate vision and according to the cost of MIOLs. Second, the grade of the PCO would be a more objective and quantitative index than the incidence of Nd: YAG laser capsulotomy. The retrospective design of our study is considered another limitation, so prospective studies should be performed to avoid potential bias.

## Conclusion

Our study indicates no significant advantage of polishing on the anterior capsule to decrease the rate of Nd: YAG laser capsulotomy after phacoemulsification in different MIOLs. ACP might not be a routine choice for ophthalmologists when attempting to reduce PCO. However, eyes with diffractive MIOLs (AT LISA tri 839MP) had a higher incidence of Nd: YAG capsulotomies.

## Data Availability Statement

The original contributions presented in the study are included in the article/supplementary material, further inquiries can be directed to the corresponding author.

## Ethics Statement

The studies involving human participants were reviewed and approved by the Ethics Committee of Qingdao Eye Hospital of Shandong First Medical University. The patients/participants provided their written informed consent to participate in this study. Written informed consent was obtained from the individual(s) for the publication of any potentially identifiable images or data included in this article.

## Author Contributions

LL and XW were the major contributors to the experimental design and drafting of the manuscript. HL and HB analyzed and interpreted the collected data. DL and YH contributed to the study concept and design. All authors reviewed and approved the final manuscript.

## Conflict of Interest

The authors declare that the research was conducted in the absence of any commercial or financial relationships that could be construed as a potential conflict of interest.

## Publisher’s Note

All claims expressed in this article are solely those of the authors and do not necessarily represent those of their affiliated organizations, or those of the publisher, the editors and the reviewers. Any product that may be evaluated in this article, or claim that may be made by its manufacturer, is not guaranteed or endorsed by the publisher.
